# Complex health care interventions: Characteristics relevant for ethical analysis in health technology assessment

**DOI:** 10.3205/hta000124

**Published:** 2016-03-24

**Authors:** Kristin Bakke Lysdahl, Bjørn Hofmann

**Affiliations:** 1 Centre for Medical Ethics, University of Oslo, Oslo, Norway; 2 The Norwegian University of Science and Technology, Gjøvik, Norway

**Keywords:** complex intervention, complex technology, ethics, ethical analysis, health technology, health technology assessment

## Abstract

Complexity entails methodological challenges in assessing health care interventions. In order to address these challenges, a series of characteristics of complexity have been identified in the Health Technology Assessment (HTA) literature. These characteristics are primarily identified and developed to facilitate effectiveness, safety, and cost-effectiveness analysis. However, ethics is also a constitutive part of HTA, and it is not given that the conceptions of complexity that appears relevant for effectiveness, safety, and cost-effectiveness analysis are also relevant and directly applicable for ethical analysis in HTA. The objective of this article is therefore to identify and elaborate a set of key characteristics of complex health care interventions relevant for addressing ethical aspects in HTA. We start by investigating the relevance of the characteristics of complex interventions, as defined in the HTA literature. Most aspects of complexity found to be important when assessing effectiveness, safety, and efficiency turn out also to be relevant when assessing ethical issues of a given health technology. However, the importance and relevance of the complexity characteristics may differ when addressing ethical issues rather than effectiveness. Moreover, the moral challenges of a health care intervention may themselves contribute to the complexity. After identifying and analysing existing conceptions of complexity, we synthesise a set of five key characteristics of complexity for addressing ethical aspects in HTA: 1) multiple and changing perspectives, 2) indeterminate phenomena, 3) uncertain causality, 4) unpredictable outcome, and 5) ethical complexity. This may serve as an analytic tool in addressing ethical issues in HTA of complex interventions.

## Introduction

The assessment of health care technology has been expanded towards ever more complex interventions, which poses a range of practical and methodological challenges. Examples of complex interventions are stroke units, strategies for implementing guidelines, community-based programmes to prevent heart disease, school-based interventions to reduce smoking or avoid teenage pregnancy, and cognitive behavioural therapy for depression [1]. To understand what makes such interventions challenging in Health Technology Assessment (HTA), we first need to know what we mean by *complex health care intervention*. The term need to be explained in a few more words than a simple definition can provide. The examples imply that *intervention* is defined broadly as a catch-all term, that includes clinical treatment, health care programs, health services delivery, and health policy [2]. It may be more difficult to grasp the *complexity* that connects these examples, as it is difficult even for researchers to agree on how to conceptualize complexity [3]. 

The origin of the word complexity is the Latin *complexus*, where *com* means ‘together’ and *plectere* means ‘to wave’ or ‘braid’, i.e. it has to do with “how things are connected with each other, and how these interactions work together” [4]. One key feature of interaction between connected things is a lack of predictability, and uncertainty and ambiguity is what distinguishes complexity from complicatedness, partly due to the capacity to adapt to changing conditions [5]. Sturmberg and Marine provide a simple illustration of the difference: an airplane is complicated, but not complex, because we can trust that the many parts work together in a predictable way while a “children’s birthday is complex because the many actors behave rather unpredictably and the behaviour of a party can change abruptly – unforeseen or unpredictably – with any minor changes in its environment” [4]. 

In health care, complexity is a feature of many aspects: the *intervention*, the *disease*, the *patient-group*, the *system*, and the *context* in which the intervention is implemented. In addition, the *investigation* of interventions can be described as complex, and place obligations on the health technology assessor. Complexity is rarely a feature of only one of these aspects, as the complexity of aspects are often interlinked. A few examples are given in the following to illustrate and explain complexity as a feature of various aspects. 

One important aspect of complex health care interventions is the central position of acting persons (rather than devices etc.), which interact with each other and the environment. The behaviour of others and the *context* influences people’s choices and actions. For instance, in *interventions* like stop smoking campaigns the smoker’s actions depend on e.g. their health status, and the actions of the clinical staff are guided by e.g. the trial protocol [6]. Likewise, the environment influences the cooperation between professional groups and care providers [7]. Shiell et al. argue that complexity is a property of a health care *system*, which is “adaptive to changes in its local environment, is composed of other complex systems (for example, the human body), and behaves in a non-linear fashion (change in outcome is not proportional to change in input)” [8].

A specific *disease*, like diabetes (type 1), can also be described as complex because the outcome is unpredictable and highly dependent on the patient’s behaviour, characteristics, preferences, and context. Additionally, the disease can be defined in many ways, a variety of treatment options exist, and a wide range of health professionals, who are supposed to interact, are involved. Complexity can further increase in cases of multiple co-morbidities, as the different treatments and other important concerns may be interacting or conflicting. Palliative care services are an example of the latter.

How we *investigate* complex interventions can also be described as complex, but Petticrew et al. [9] emphasise that investigation of a complex interventions does not always require complex approaches. Narrowly focused research questions are legitimate, and could be investigated in parallel with complex analysis, but the researcher should never ignore aspects of complexity in the intervention being assessed [9]. Complex interventions can also entail specific obligations on the reviewers, e.g. to examine how the influence and power of different parties affect implementation [2]. 

The methodological challenges with assessing effectiveness, safety, and cost-effectiveness of complex interventions have recently received much interest in the HTA literature [9]. One important aspect of these challenges is the combining of natural and social sciences. This interest has resulted in a series of articles on implications of complexity of interventions for systematic reviews (e.g. in the November issue 2013 of the Journal of Clinical Epidemiology). Hence, there exist useful overviews of aspects of complexity to address when assessing safety, effectiveness, and cost-effectiveness. However, specific challenges may occur in ethical analysis in HTA as well, and the aspects of complexity relevant in systematic reviews may be more or less relevant in ethical analysis. Moreover, complexity may pose specific (additional) moral challenges for ethical analyses in HTA, and moral issues may themselves add to the complexity.

The aim of this study is to establish a set of characteristics of complex health care interventions relevant for addressing ethical aspects in HTA. In other words, characteristics that should be taken into account in ethical analysis of complex health interventions, independent of the choice of methodological approach for the analysis. The differences between the many approaches for ethical analysis [10] is not of interest here. Our point of departure is that they all aim to illuminate moral impact of implementing health technologies, and embedded values in the technology that may challenge moral norms and values in society [11]. To establish this set of characteristics we will first assess whether or not the characteristics of complex interventions described in the literature on (systematic reviews in) HTA are relevant for ethical analysis. Then, we will identify and elaborate the key characteristics of complex interventions most relevant for ethical analysis in HTA. Finally, we will indicate some implications of these characteristics for ethical analysis in HTA and beyond. 

## Identified characteristics of complex interventions in the HTA literature

Two notable publications in the HTA literature provide comprehensive lists of characteristics of complexity. First, the framework for development and evaluation of RCTs for complex interventions provided by the UK Medical Research Council (MRC), which lists the following characteristics (direct quotes except for the numbering) [12]:

1. Number of interacting components within the experimental and control interventions
2. Number and difficulty of behaviours required by those delivering or receiving the intervention
3. Number of groups or organisational levels targeted by the intervention
4. Number and variability of outcomes
5. Degree of flexibility or tailoring of the intervention permitted [12]

Second, the work of Petticrew and colleagues extend the list with the following 7 characteristics (direct quotes except for the order and numbering) [9]:

1. Self-organization, adaptivity, and evolution over time
2. Nonlinear relationships (cannot be arranged along a simple input–output line); phase changes
3. Feedback loops, (e.g., where changes in behavior create the conditions for behavior to change further and where uptake in cycling results in more cyclists, which means that cycling becomes the norm, encouraging more people to take up cycling)
4. Synergy between components, and does the program have symbolic value over and above its operational components?
5. Multiple mediators and moderators of effect such as the background characteristics and environment of the patient
6. Connectivity, where individual components of an intervention are linked together in a system, so they influence each other
7. Interaction with context and the capability created from this interaction; very susceptible to effect of different contexts (e.g., policy timing, organizational culture and leadership, resource allocation, staffing levels and capabilities, interpersonal relationships)

The work of the MRC has been influential and is included in the summary of the literature in the field by Petticrew et al. [9]. While the MRC’s report focuses on the characteristics of the intervention itself, Petticrew and colleagues’ review adds characteristics of the intervention’s causal pathway. (Characteristics of the intervention itself includes number 1,3, 5 and 6, while the others characterise the causal pathway [9], except for number 4 which was not included in the overview from Petticrew et al.).

Keeping in mind the centrality of unpredictability in complexity, one may, of course, ask whether some of the characteristics are justified. The MRC characteristics focus on numbers and difficulties, which make interventions more complicated, but not necessarily more complex. For instance, “Number of interacting components” (no.1) is not a characteristic of complexity if the interactions are perfectly predictable. A reason for including them can be that complicated aspects of these characteristics can increase the likelihood of unpredictability in the specific context of health care. The unpredictability entailed in other characteristics is more evident, for instance in “Self-organization, adaptivity, and evolution over time” (no. 6). 

The characteristics of complexity are identified mainly because they challenge the relevance or usefulness of existing methods for effectiveness and cost-effectiveness assessment in HTA. One example is the difficulty of integrating heterogeneous evidence, i.e. including data from different (quantitative and qualitative) study designs. However, such issues are not as pertinent in ethical analysis, where a range of information sources (qualitative and theoretical research, policy documents etc.) are regularly included without any difficulties. However, as our analysis will show, several of these characteristics can still be relevant for ethical analysis for other reasons.

## What is the relevance of the identified characteristics of complexity for ethical analysis in HTA?

In the following we explore the complexity characteristics further and give a few examples of ethical issues they can raise. As some of the characteristics of complexity listed above are ethically relevant in similar ways, we will assess them together under the same subheading (below). We use exact quotes in the subheadings to make the link to the original characteristics clear; otherwise we adjust the terminology to better fit an ethical analysis. Throughout the analysis, identified and conceptualized overarching aspects are highlighted in **bold letters**, in order to make the steps towards a synthesis of ethically relevant complexity characteristics transparent. 

*1. Number of interacting components*

A health care intervention may consist of different kind of components; material (device), procedural, theoretical, organisational, stakeholders etc. From an ethical point of view, a number of actors/stakeholders may be particularity important because it means that the intervention and its outcome can be influenced by individual human phenomena, “how people see, understand and experience the intervention in relation to their bodies, beliefs, attitudes, knowledge, skills and values” [13]. Besides the human features, the parts that form a complex intervention can be e.g. material, theoretical, social, and procedural in nature [13]. E.g. a complex intervention like palliative care is diverse in terms of elements: content (e.g. pain relief, psychological support etc.), theoretical basis (e.g. holistic vs. task-oriented care), providers’ professions, and setting (e.g. hospital, home care). The many elements (human and other) mean that the intervention can be viewed from **multiple and changing perspectives**, which is relevant for ethical analysis because it may cause a conflict of interest, and represent a challenge to democracy and justice. How should the different perspectives be reflected in a fair and transparent manner? Moreover, the interaction between components may call for awareness of power asymmetries, and the importance of trust.

* 2. Number and difficulty of behaviours required by those delivering or receiving the intervention*

Various interventions that require variable and dynamic behaviours from actors indicate that the intervention is not well defined/delimited, i.e. an **indeterminate phenomena**. If different actors understand the intervention (and its elements) differently and find their tasks difficult, this will influence their actions, which in turn constitute components of the intervention. Again, palliative care can serve as an example, where e.g. decision-making can be challenging due to patients’ reduced decision-making capacity, professional (culture dependent) reluctance towards withdrawing/withholding aggressive or futile treatment, and finally, difficult cooperative tasks between the many health providers involved. This example illustrates the relevance of ethical questions like (patient and professional) autonomy, responsibility diffusion, accompanied by questions of overtreatment, futility and resource allocation.

*3. Number of groups or organisational levels targeted by the intervention*

Different patient groups may be potential users of a set of single technologies/interventions, which together constitutes a complex technology. This adds to complexity through **multiple and changing perspectives** (see point 1). This calls for awareness of transdisciplinarity [14], and the interconnections between systems and the single technologies involved. A relevant ethical question is “determining responsibility and liability for errors and damages”, which is much more difficult in complex interventions [15]. Besides, one can ask whether the intervention is equally feasible, accessible and beneficial to all targeted groups.

*4. Number and variability of outcomes*

There may be many, variable, unexpected, new, and emergent outcomes, i.e. **outcomes** are **unpredictable**. This means that the type, as well as the size and the time of outcomes are uncertain. Unintended or unforeseen uses and outcomes appear to be typical for health technologies [16]. As fairly simple technologies, such as blood pressure regulating drugs, may have unintended outcomes (on erectile dysfunction), this becomes even more so for complex technologies. Accordingly, we cannot be sure about benefits, risks and costs of the intervention, which are crucial for applying ethical principles such as beneficence, nonmaleficence, and justice [17], as well as equity and solidarity. Besides, it is possible that the outcomes may disfavour or harm particularly vulnerable groups of people, threaten dignity, and cause stigmatisation. 

*5. Degree of flexibility or tailoring of the intervention permitted and 6. Self-organisation, adaptivity, and evolution over time*

These two characteristics (flexibility/tailoring and self-organisation/adaptivity) point to an intervention that is hard to delimit and define as its characteristics can be temporal and unpredictable, i.e. it can be described as an **indeterminate phenomenon**. This indeterminacy, e.g. dependent on patient preferences, context characteristics, health policy issues etc., is ethically relevant because it may affect the aim, structure, and outcome of the intervention. Moreover, we may raise the question of the moral legitimacy of (alterative) use of the intervention, and acceptability of the tailoring process (e.g. the role of patient preferences).

*7. Nonlinearity; phase changes, 8. Feedback loops, 9. Synergy and symbolic value, 10. Mediators and moderators, and 12. Interaction with context*

In these 5 characteristics the focus is moved from variation and uncertainties in outcome (see point 4), to causal pathways that may bring about this unpredictability, which we may label **uncertain causality**. E.g. non-linear interactions means that “small, random changes can lead to large changes in that system” [14], and the symbolic value of an intervention may alter the implementation process substantially. Moreover, unclear causality may result from uncertainty in several ways, e.g., in terms of known probabilities for given outcomes (risk), unknown probabilities for given outcomes (uncertainty), unknown outcomes and hence, unknown probabilities, (ignorance) [18]. Additionally, some relationships are uncertain due to unclear or value-laden conceptions and classifications (indeterminacy). Beyond the methodological challenges they pose for effectiveness analysis, all these types of uncertainty are relevant for the ethical analysis. E.g. they pose challenges with respect to the value of risk (risk aversion) and knowledge (value of knowledge). They are also relevant because they illustrates the value-ladenness of methodological choices in HTA and of the social commitment involved. E.g. not taking moral, socio-cultural, professional, or legal values and norms into account in a specific context for the intervention in the planning and execution of the HTA, increases the risk of causing harm and injustice.

*11. Connectivity, where individual components of an intervention are linked together in a system, so they influence each other*

Connectivity between components are ethically relevant for similar reasons as given in point 1 – **multiple and changing perspectives**, i.e. when components are interacting, linked together and influence each other, some key issues appear: (a)symmetries of power, free will, control and decision-making. These issues are in turn particularly relevant for normative questions of responsibilities, blame, and acceptance of risk. 

In summary, several characteristics of complexity identified for the assessment of complex interventions in the HTA literature are relevant also for ethical analysis in HTA, and for a variety of ethical reasons. Throughout the analysis, we have identified four overacting characteristics considered relevant for ethical analysis in HTA. In the following, we suggest adding the characteristic **ethical complexity**, which appears to be relevant for assessing ethical aspects of complex interventions. 

## Ethical complexity characteristics

As revealed above, various forms of complexity may imply ethical challenges, i.e., complexity of a health care intervention may serve as a source of ethical questions the analysis need to address. However, ethical aspects may themselves contribute to an intervention’s complexity, when ethical complex questions are involved. There are many ways this can happen. Here we mention only two circumstances that can add to such **ethical complexity**. The two aspects of ethical complexity described below should not be understood as mutually exclusive in the sense that they cannot both appear when assessing a health care intervention. Still they represent to different ways ethical issues can contribute to complexity of an intervention. 

The first aspect of ethical complexity regards interventions where fundamental moral or socio-cultural values are at stake, e.g. the intervention challenges people’s beliefs and understanding of the value, integrity, or dignity of human life. In such cases, the potential for public engagement, debate and controversy is high, and complexity-thinking should accordingly include awareness of “broader societal involvement” [14]. There are many examples of rather simple interventions (e.g., with a low number of components etc.), that have the potential to cause (more or less expected) controversy: non-invasive prenatal diagnosis, bariatric surgery, expensive cancer drugs et cetera. Cochlear implants exemplify an intervention that caused considerable debate in the deaf community. This unexpected scepticism was first identified as a concern for upholding Deaf culture and sign language. Later the issue of a problematic rehabilitation process and the harmful effects related to the idea of normalisation appeared [19]. The key point here is that the case illustrates how socio-cultural and ethical values may give rise to complexity in the decision-making process following the HTA. Hence, it is important that ethical analysis in HTA uncovers potential fundamental ethical, social, and cultural values at stake, and contributes to the handling of conflicting concerns.

The second aspect of ethical complexity regards intervention where contradictions between basic (ethical) principles are embedded. Implementation of new diagnostic technology may serve as an example. The development of radiological technologies has increased the ability to detect (and subsequently treat) people’s physical diseases. At the same time, this technology is also increasingly used to psychologically comfort people’s health anxiety. If one believes that physical and mental diseases belong to different spheres, governed by different principles, or that technology developed for diagnostics does not automatically have therapeutic (anxiolytic) functions, one is faced with an antitomy. Antitomies are contradictions between two apparently equally valid principles, which are hard to solve as they require shifting references, perspectives, or paradigms [20]. Similarly, one can argue that implementation of clinical guidelines involves a contradiction between two profound ethical principles: (professional) autonomy and heteronomy. Guidelines pose restrictions on the professionals’ actions (heteronomy) and at the same time facilitates the professionals’ (autonomous) actions towards the goals of health care. Ethical complexity from contradictions between basic principles also includes contradictions “between inferences correctly drawn from such principles” [20].

Some contradictions may seem unresolvable (aporias), such as e.g. the moral status of the foetus in the assessment of prenatal tests or reproductive technologies. The ethical analysis should elucidate possible contradictions that are hard to solve/insolvable and differentiate these from contractions that are (more easily) resolvable: e.g. those resolvable through clarification of ambiguity of concepts. This will inform whether and how challenges of contractions can be met. Such a task may require philosophical qualifications.

## Summary of complexity characteristics

The investigation indicates that it is possible to amend, synthesise, and elaborate the many characteristics of complexity into some general/overarching characteristics relevant for ethical analysis in HTA. Table 1 (Tab. 1) lists five main characteristics, with a short explanation based on the analysis above. In addition, we indicate some implications of these characteristics for ethical analysis in HTA, which might be useful when assessing/choosing the ethical approach.

## Discussion

The nature of complex health care interventions makes it challenging to define, characterise and delimit. It can in fact be argued that most health care interventions are complex to some extent, since hospitals and primary care services are examples of complex systems [6]. However, several characteristics of particularly high-level complex interventions are identified in the literature. In this analysis, we synthesised a set of five characteristics considered relevant for ethical analysis in HTA: multiple and changing perspectives, indeterminate phenomena, uncertain causality, unpredictable outcome and ethical complexity. 

Clearly, the inclusion of other publications on the complexity of health care interventions could have given different outcomes. However, an initial broader literature search revealed that the two key articles covered most frequently, identified characteristics of complexity. Moreover, we do not claim that the five characteristics are mutually exclusive criteria of complexity. Surely, they can be interrelated and overlapping. Ethical complexity can be influenced by, for instance, the multiple perspectives, indeterminacy and unpredictability involved.

Complexity characteristics of health care interventions can be ethically relevant in several ways, and depend on the ethical approach applied. Hence, regarding completeness of the analyses, it should be noted that we neither aimed to identify *all* reasons why a specific characteristic of complexity is ethically relevant, nor to provide an exhaustive overview of implications for ethical analysis in HTA (Table 1 (Tab. 1)). Rather, we aimed to provide examples sufficient to illustrate the ethical relevance, and describe some obvious implications that illustrate the demands complex interventions pose upon the ethical approaches. 

We also acknowledge that the term ‘ethical complexity’ may appear strange or artificial, as most ethical aspects can be conceived of as complex. However, we think that in the context of HTA it makes sense to label a certain type of complexity as ‘ethical’. One reason for this is to generate higher awareness of ethical issues in HTA. Public health care interventions involving social determinants of health (like unemployment, education, social network, lifestyle factors, access to health services etc.) are an interesting example. The HTA research on social determinants of health is clearly initiated by the ethical concern of equity [21], [22], and the relevant interventions will score high on many complex characteristics (can be hard to define, can cause unintended, unpredictable outcome etc.). The question is whether these interventions also can be characterised as *ethically complex*. On the one hand, equity is clearly the ethical concern, and there may be no evident contradictions between the principle of equity and other basic (ethical) principles. On the other hand, the understanding of equity, fairness and justice varies. Moreover, peoples’ integrity may be violated by implementation of an intervention that e.g. is aiming to change their lifestyle. Consequently, interventions involving social determinants can potentially cause public controversy, and be characterised as ethically complex. 

The aspects of complexity identified as relevant for the assessment of complex health care interventions may of course be relevant for addressing ethical issues in health care in general. All 5 characteristics appear important for ethical analysis in health care settings in general. The reason this article is restricted to ethical analysis in HTA, is that this is where a method for ethical analysis is especially needed [10], [23].

The analysis shows that the complexity of an invention may be ethically relevant in many ways, and indicates that this poses high demands on the ethical analysis in HTA. Hence, we consider the analysis a warranted first step in investigations of how to deal with complex interventions in analyses of ethical issues in HTA. How to implement the characteristics in the assessment of complex interventions in practice is the next step. Here, we only suggest a synthesised set of complexity characteristics that are important to have in mind when addressing ethical issues in the HTA of complex interventions, for instance as a tool for assessing the applicability of existing ethical approaches.

## Conclusion

This article has taken as its point of departure the importance of acknowledging the complexity of health care interventions in HTA. The aspects of complexity shown in the literature to be relevant when assessing effectiveness, safety, and efficiency, are in this article also shown to be relevant when assessing ethical issues of a given health technology. However, these concerns are altered when ethical issues are addressed, and certain aspects of complexity are specifically ethically relevant, compared to when addressing effectiveness, safety, and efficiency issues. The synthesised set of key characteristics of complexity for addressing ethical aspects in HTA are: 1) multiple and changing perspectives, 2) indeterminate phenomena, 3) uncertain causality, 4) unpredictable outcome, and 5) ethical complexity. This may serve as an analytic tool in addressing ethical issues in HTA of complex health care interventions.

## Notes

### Acknowledgement 

This paper is written within the research project INTEGRATE HTA funded by the European Commission (Grant agreement no. 306141). We would like to thank members of the project team: Eva Rehfuess and James B. Chilcott for increasing our understanding of complexity, and Wija Oortwijn, Louise Brereton and Pietro Refolo for feedback on early drafts of the manuscript.

### Competing interests

The authors declare that they have no competing interests.

## Figures and Tables

**Table 1:**
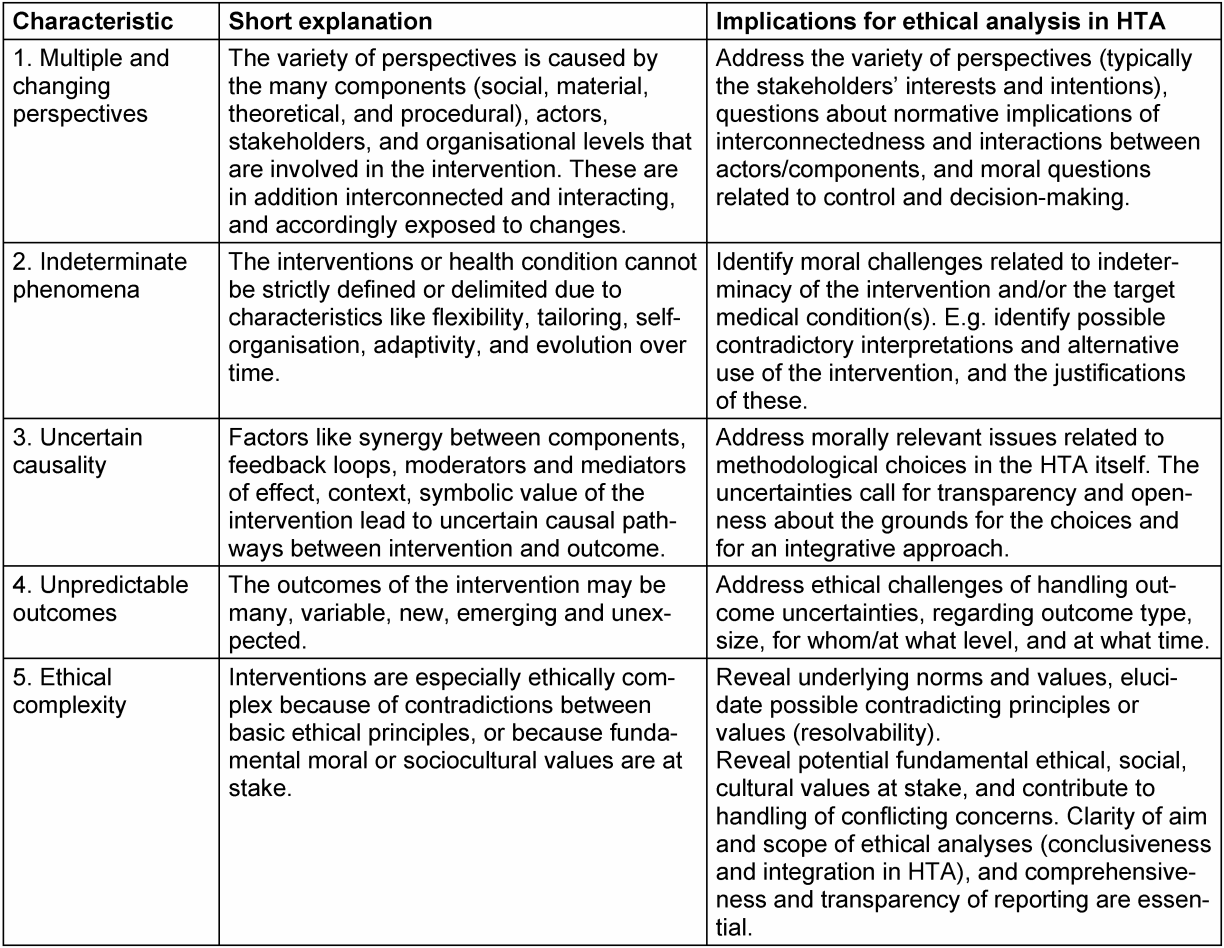
Summary of most relevant characteristics of complexity for addressing moral issues in HTA and some implications for ethics analysis in HTA

## References

[1] Campbell M, Fitzpatrick R, Haines A, Kinmonth AL, Sandercock P, Spiegelhalter D, Tyrer P (2000). Framework for design and evaluation of complex interventions to improve health. BMJ.

[2] Pawson R, Greenhalgh T, Harvey G, Walshe K (2005). Realist review – a new method of systematic review designed for complex policy interventions. J Health Serv Res Policy.

[3] Wong G (2013). Is complexity just too complex?. J Clin Epidemiol.

[4] Sturmberg JP, Martin CM (2013). Handbook of Systems and Complexity in Health.

[5] Glouberman S, Zimmerman B (2002). Complicated and complex systems: what would successful reform of medicare look like?.

[6] Shepperd S, Lewin S, Straus S, Clarke M, Eccles MP, Fitzpatrick R, Wong G, Sheikh A (2009). Can we systematically review studies that evaluate complex interventions?. PLoS Med.

[7] Hasson H (2010). Systematic evaluation of implementation fidelity of complex interventions in health and social care. Implement Sci.

[8] Shiell A, Hawe P, Gold L (2008). Complex interventions or complex systems? Implications for health economic evaluation. BMJ.

[9] Petticrew M, Anderson L, Elder R, Grimshaw J, Hopkins D, Hahn R, Krause L, Kristjansson E, Mercer S, Sipe T, Tugwell P, Ueffing E, Waters E, Welch V (2013). Complex interventions and their implications for systematic reviews: a pragmatic approach. J Clin Epidemiol.

[10] Assasi N, Schwartz L, Tarride JE, Campbell K, Goeree R (2014). Methodological guidance documents for evaluation of ethical considerations in health technology assessment: a systematic review. Expert Rev Pharmacoecon Outcomes Res.

[11] Saarni SI, Hofmann B, Lampe K, Lühmann D, Mäkelä M, Velasco-Garrido M, Autti-Rämö I (2008). Ethical analysis to improve decision-making on health technologies. Bull World Health Organ.

[12] Craig P, Dieppe P, Macintyre S, Michie S, Nazareth I, Petticrew M, Medical Research Council Guidance (2008). Developing and evaluating complex interventions: the new Medical Research Council guidance. BMJ.

[13] Clark AM (2013). What are the components of complex interventions in healthcare? Theorizing approaches to parts, powers and the whole intervention. Soc Sci Med.

[14] Lessard C (2007). Complexity and reflexivity: two important issues for economic evaluation in health care. Soc Sci Med.

[15] Manzeschke A, Weber K, Fangerau H, Rother E, Quack F, Dengler K (2013). An ethical evaluation of telemedicine applications must consider four major aspects – a comment on Kidholm et Al. Int J Technol Assess Health Care.

[16] Hofmann B (2005). Toward a procedure for integrating moral issues in health technology assessment. Int J Technol Assess Health Care.

[17] Beauchamp TL, Childress JF (2001). Principles of biomedical ethics.

[18] Wynne B (1992). Uncertainty and environmental learning: Reconceiving science and policy in the preventive paradigm. Glob Environ Change.

[19] Kermit P (2012). Enhancement technology and outcomes: what professionals and researchers can learn from those skeptical about cochlear implants. Health Care Anal.

[20] Hofmann B (2001). The paradox of health care. Health Care Anal.

[21] Bambra C, Gibson M, Sowden A, Wright K, Whitehead M, Petticrew M (2010). Tackling the wider social determinants of health and health inequalities: evidence from systematic reviews. J Epidemiol Community Health.

[22] Tugwell P, Petticrew M, Kristjansson E, Welch V, Ueffing E, Waters E, Bonnefoy J, Morgan A, Doohan E, Kelly MP (2010). Assessing equity in systematic reviews: realising the recommendations of the Commission on Social Determinants of Health. BMJ.

[23] Hofmann BM (2008). Why ethics should be part of health technology assessment. Int J Technol Assess Health Care.

